# Greater occipital nerve iatrogenic neuroma after cervical arthrodesis: high-frequency ultrasound findings and ultrasound-guided management

**DOI:** 10.1007/s00256-026-05362-5

**Published:** 2026-08-31

**Authors:** Cláudia Fontan, Carolina Ávila de Almeida, Danielle Martiniano da Silva Rodrigues, Victor Alfredo de Santana Silva, Clarissa Canella

**Affiliations:** 1 Universidade Federal de Pernambuco (UFPE), Recife, Brazil; 2 Universidade Federal Do Estado do Rio de Janeiro (UNIRIO), Rio de Janeiro, Brazil; 3 Universidade Federal Fluminense (UFF), Niterói, Brazil; 4 Universidade Federal do Rio de Janeiro (UFRJ), Rio de Janeiro, RJ Brazil

**Keywords:** Occipital neuralgia, Cervical arthrodesis, Ultrasonography, Peripheral nerve, Iatrogenic injury, Neuroma

## Abstract

Persistent occipital headache after cervical arthrodesis is a disabling complication, often due to iatrogenic injury to the greater or lesser occipital nerves. Clinical overlap with primary headaches may delay diagnosis. High-resolution ultrasound (HRUS) has emerged as a reliable tool for evaluating superficial peripheral nerves and postoperative changes. We report a case illustrating its diagnostic value in occipital nerve injury, with a focused review of sonoanatomy. A 45-year-old woman developed refractory bilateral occipital pain consistent with occipital neuralgia after cervical arthrodesis for disc herniation. HRUS (24 MHz) using a standardized protocol revealed postsurgical changes and focal fusiform thickening with loss of fascicular architecture of the right greater occipital nerve at its muscular perforation site, consistent with iatrogenic neuroma. These findings guided targeted management. This case underscores the diagnostic challenge of postsurgical occipital neuralgia and the utility of HRUS. Knowledge of occipital nerve sonoanatomy and its relationship to adjacent cervical muscles is essential. HRUS enables real-time evaluation of nerve morphology and continuity, detecting focal thickening often missed by other imaging methods. HRUS allowed diagnosis and percutaneous treatment of greater occipital nerve neuroma in this patient.

## Introduction

Persistent headache is a recognized and potentially disabling complication following cervical spine surgery, particularly posterior arthrodesis. This entity is frequently attributed to iatrogenic injury of the occipital nerves, whose superficial course and muscular perforation points render them vulnerable during posterior approaches [1, 2]. Injury to the greater (GON), lesser, or third occipital nerves may result in occipital neuralgia, classically characterized by paroxysmal, lancinating pain in their respective sensory distributions [3].

The differential diagnosis of headache after cervical arthrodesis is challenging, as clinical manifestations often overlap with primary headache disorders, including migraine and tension-type headache [3]. Although peripheral nerve injury is a recognized complication of cervical procedures, the specific pathophysiological mechanisms linking posterior cervical arthrodesis to occipital neuralgia remain insufficiently characterized [4, 5].

High-resolution ultrasound (HRUS) has emerged as a first-line imaging modality for the evaluation of superficial peripheral nerve disorders [5, 6]. In cases of suspected occipital neuralgia, HRUS facilitates direct identification of the greater and lesser occipital nerves along their anatomical course and enables detection of neuromas, entrapment, or post-surgical scarring [7].

Despite these advantages, the role of HRUS in the specific setting of post–cervical arthrodesis headache remains underreported. This case report reviews the relevant sonoanatomy and illustrates the diagnostic value of neuromuscular ultrasound in identifying an occipital nerve neuroma as the cause of refractory post-arthrodesis headache. A systematic scanning protocol is also illustrated to support reproducibility and broader clinical implementation.

## Case report

A 45-year-old woman was referred for HRUS evaluation due to persistent occipital pain following cervical arthrodesis. The patient’s clinical history began approximately 1 year earlier with development of left upper limb weakness and impaired abduction. Imaging demonstrated cervical disc herniation, and the patient subsequently underwent posterior cervical arthrodesis.

In the postoperative period, the patient developed severe bilateral occipital headache with clinical features consistent with occipital neuralgia. The pain was bilateral but consistently more intense on the right side on physical examination. No definitive etiology was established, and symptoms were refractory to conservative management.

HRUS examination was performed using a Canon Aplio i700 system with high-frequency linear transducers (up to 24 MHz). The study demonstrated expected post-surgical changes involving the skin, subcutaneous tissue, and posterior cervical musculature, with posterior acoustic shadowing related to metallic fixation at the level of the posterior vertebral elements. A high-resolution linear transducer (14–24 MHz) was used, with the patient in the seated position and slight cervical flexion. The greater occipital nerves (GONs) were identified bilaterally using a standardized landmark-based approach. With the transducer positioned transversely over the C2 spinous process and tilted laterally, the GON was visualized within the fascial plane between the obliquus capitis inferior (deep) and the semispinalis capitis (superficial) muscles. The nerve was traced cranially until it pierced the trapezius aponeurosis, and its caliber, echotexture, fascicular integrity, and continuity were assessed. Cross-sectional area measurements were obtained and compared with the contralateral side. The lesser occipital nerve was evaluated more laterally, along the posterior border of the sternocleidomastoid muscle. Particular attention is directed toward focal hypoechoic enlargement, fascicular disruption, discontinuity, or fusiform lesions suggestive of neuroma. Power Doppler may be used to assess hypervascularity.

**Fig. 1:**
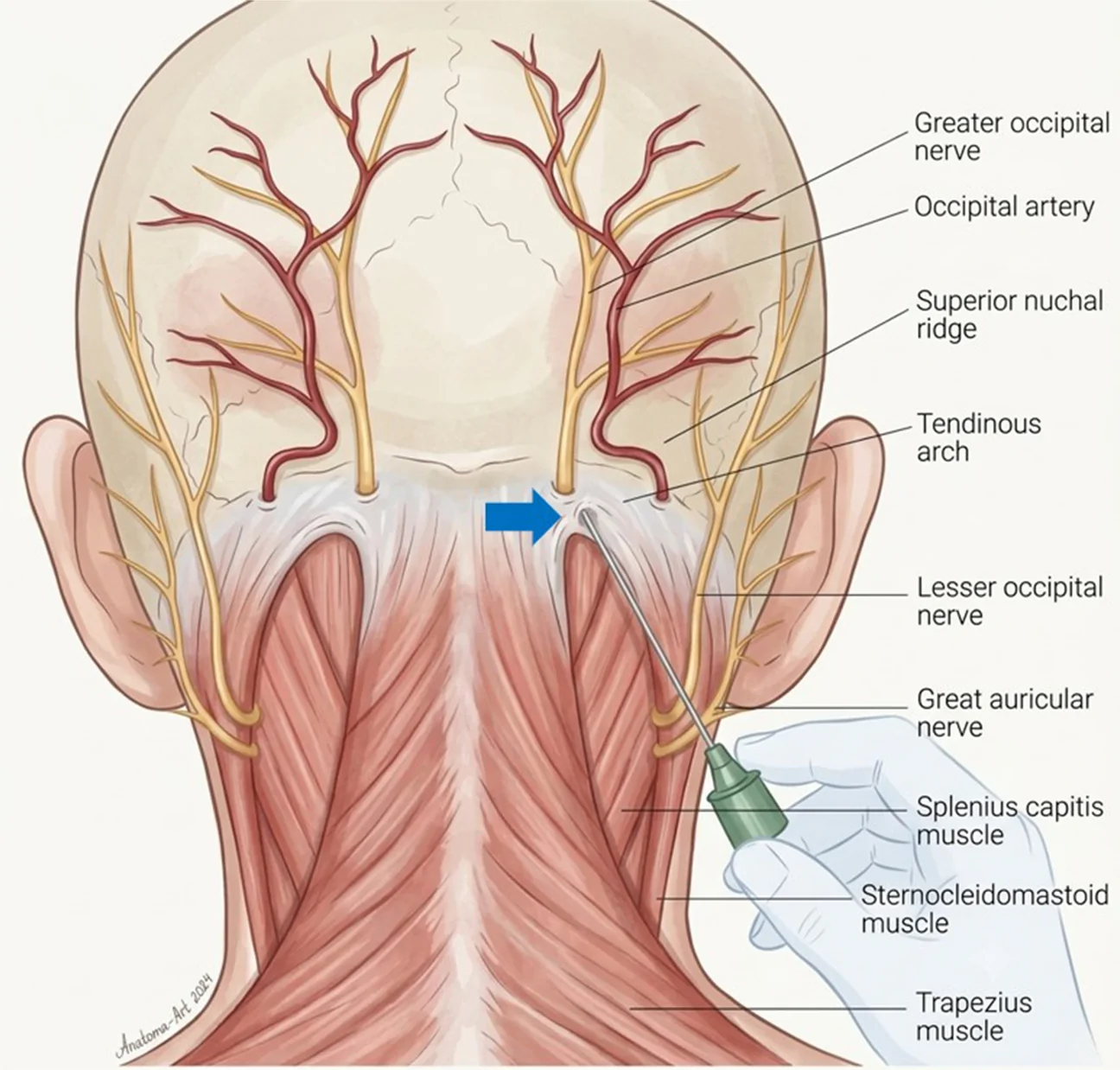
Schematic illustration of the posterior cervical region demonstrating the anatomical course of the greater occipital nerve (GON) and lesser occipital nerve (LON), their relationship with the adjacent cervical musculature, and the course of the occipital artery. Schematic representation also demonstrates the placement of greater occipital nerve block (arrow). Illustration generated using Google Gemini, based on data and visual concepts from Lee et al. [20]

**Fig. 2:**
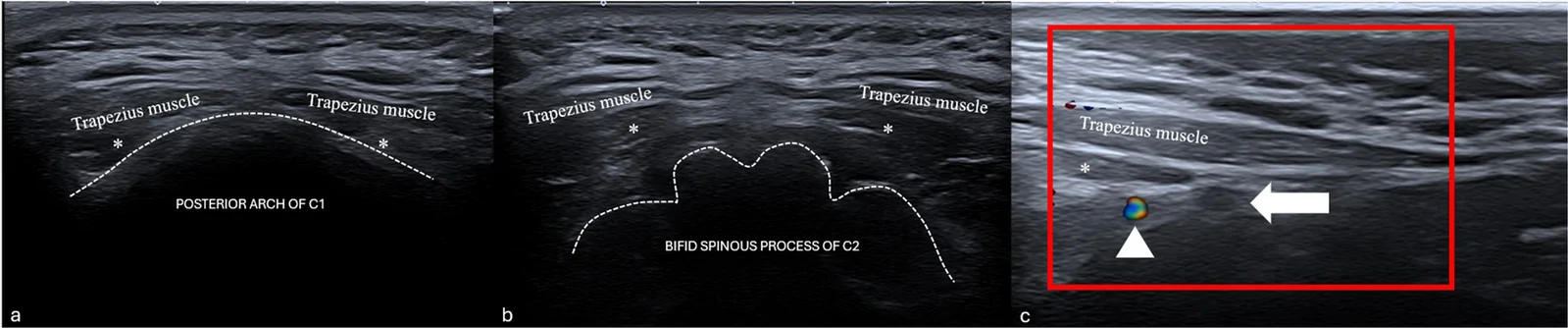
Standardized ultrasound scanning technique for identification of the greater occipital nerve (GON). **a **HRUS (24 MHz), transverse midline image of the upper cervical region demonstrating the posterior arch of C1 (solid line). **b **HRUS (24 MHz), transverse midline image immediately caudal to (**a**), showing the posterior arch and bifid spinous process of C2 (solid line), an important osseous landmark for GON localization. **c **Medium-frequency ultrasound (14 MHz) with power Doppler, transverse image obtained at the superior nuchal line demonstrating the greater occipital nerve (arrow) medial to the occipital artery (arrowhead), identified by Doppler signal. The semispinalis capitis (*) and trapezius muscles are labeled to illustrate the anatomical relationship of the nerve along its course toward the scalp

**Fig. 3:**
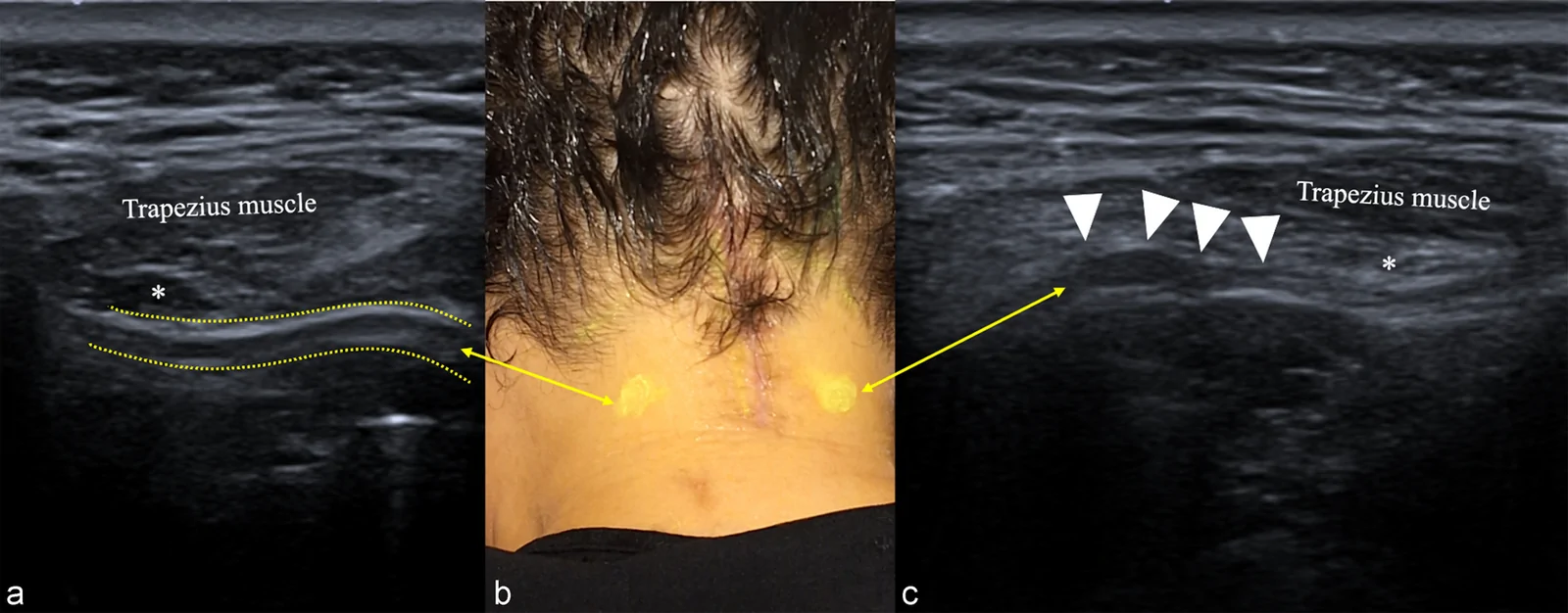
45-year-old woman with iatrogenic neuroma of the greater occipital nerve. **a **Longitudinal ultrasound image of the left greater occipital nerve (between the dotted outline) demonstrating normal morphology, with tubular configuration, hypoechoic background, and preservation of the internal parallel fibrillar echotexture, located below the trapezius muscle (*). **b **Clinical photograph showing yellow markings indicating the surface projection of the greater occipital nerve and the region corresponding to the examined nerve segment. The transducer was positioned longitudinally on the skin surface, with a slight inclination to follow the path of the greater occipital nerve. **c **Longitudinal ultrasound image of the right greater occipital nerve (arrowheads) revealing marked hypoechoic enlargement, loss of normal fibrillar architecture, and irregular margins, consistent with iatrogenic neuroma

The greater occipital nerves (GONs) were identified bilaterally using a standardized landmark-based protocol (described below) (Fig. 1). Both nerves demonstrated mild diffuse thickening along their course. On the right side, a focal hypoechoic enlargement was observed, associated with loss of normal fascicular architecture and partial structural discontinuity. The lesion had fusiform morphology and was consistent with an iatrogenic terminal neuroma (Figs. 2 and 3). The abnormality corresponded precisely to the patient’s maximal pain point on clinical examination. The left GON demonstrated no focal structural abnormalities.

Following the ultrasound findings, the patient underwent ultrasound-guided greater occipital nerve block with local anesthetic and corticosteroid. The procedure resulted in significant short-term pain relief, supporting the diagnosis of occipital neuralgia secondary to traumatic neuroma. The patient experienced substantial pain relief following the initial infiltration, but symptoms recurred approximately three months later, prompting a second ultrasound-guided infiltration that again resulted in substantial pain relief. Long-term follow-up data remain limited. Histopathological confirmation was not obtained, and the diagnosis was established based on the correlation between clinical presentation and characteristic ultrasound findings.

## Discussion

This case highlights the diagnostic value of HRUS in a patient with chronic refractory headache following cervical arthrodesis, in whom a GON neuroma was identified as the underlying etiology [1]. Persistent postoperative occipital pain is frequently attributed to occipital neuralgia (ON), yet establishing a precise structural diagnosis remains challenging, particularly when symptoms overlap with primary headache disorders [2, 8]. The prolonged diagnostic course in this patient underscores the limitations of clinical assessment alone in the absence of targeted imaging.

Iatrogenic neuroma formation is a recognized consequence of peripheral nerve injury, occurring when axonal regeneration is disrupted by fibrosis, transection, or mechanical obstruction, leading to disorganized neural proliferation and neurogenic pain [4]. The occipital nerves—especially the GON—are anatomically vulnerable during posterior cervical approaches due to their superficial trajectory and muscular perforation points [5]. Mechanisms of injury during arthrodesis include direct transection, traction during surgical retraction or positioning, compression by metallic fixation, and postoperative cicatricial fibrosis [2]. Previous studies have reported a normal GON cross-sectional area of approximately 2.0 ± 0.1 mm^2^ at the C1–C2 level, whereas symptomatic patients with occipital nerve entrapment demonstrate mean values of approximately 4.1 ± 2.6 mm^2^. The marked enlargement observed in our patient further supports the diagnosis of pathologic nerve involvement [9]. Although not extensively reported, occipital nerve injury is an acknowledged cause of secondary headache following upper cervical procedures [2]. In the present case, HRUS demonstrated focal hypoechoic enlargement of the right GON with loss of normal fascicular architecture and partial discontinuity—findings consistent with a terminal neuroma [1]. The imaging abnormality corresponded precisely to the patient’s pain topography, providing objective pathoanatomical confirmation [3].

HRUS offers distinct advantages in the postoperative cervical spine setting [1]. Its superior spatial resolution allows detailed assessment of small-caliber nerves and detection of subtle morphological alterations, including focal thickening and discontinuity [1]. Unlike MRI or computed tomography (CT), ultrasound is not degraded by metallic susceptibility artifacts from surgical implants, enabling accurate evaluation of soft tissues adjacent to metallic fixation [10]. Additionally, dynamic assessment and targeted transducer pressure allow real-time correlation with patient-reported pain, functioning as a sonographic Tinel sign [1]. Given its accessibility, cost-effectiveness, and absence of ionizing radiation, ultrasound is well suited for longitudinal follow-up [7, 10].

The diagnosis of a GON neuroma has direct therapeutic implications [11]. Precise sonographic localization facilitates image-guided interventions such as diagnostic nerve blocks, corticosteroid injections, hydrodissection, and pulsed radiofrequency treatment [12–16]. In refractory cases, detailed anatomical mapping provided by HRUS supports surgical planning, including neuroma resection or nerve stump relocation [9, 17]. Ultrasound guidance has also expanded the role of percutaneous peripheral nerve stimulation in the management of chronic neurogenic pain [13, 18, 19]. As emphasized in recent literature, ultrasound is increasingly evolving from a purely diagnostic modality to a comprehensive platform for both diagnosis and treatment of peripheral nerve conditions [14].

Although limited by its single-case design and lack of long-term outcome data [10], this report provides proof of concept that HRUS can identify clinically relevant occipital nerve pathology after cervical arthrodesis [10]. In the absence of histopathological confirmation, the combination of imaging findings and response to targeted treatment may serve as a practical diagnostic surrogate. Clinical improvement followed the combined local anesthetic and corticosteroid injection, limiting the ability to distinguish the relative contribution of either component to symptom relief. Prospective studies involving larger cohorts are necessary to establish the prevalence of this complication and to validate systematic screening protocols in patients with persistent postoperative headache [7, 10]. Nevertheless, this case supports consideration of high-resolution neuromuscular ultrasound as a first-line imaging modality in the evaluation of post-cervical surgery headache [1, 7].

## Conclusion

Greater occipital nerve neuroma should be considered in the differential diagnosis of persistent headache following posterior cervical arthrodesis. High-resolution ultrasound can directly identify occipital nerve abnormalities and establish a structural correlate for symptoms that may otherwise remain unexplained. This case highlights the value of neuromuscular ultrasound as a practical tool for both diagnosis and treatment planning in patients with suspected postoperative occipital neuralgia.

## Data Availability

The data supporting the findings of this study are available within the article. Additional information may be provided by the corresponding author upon reasonable request.
